# Hedgehog signalling is required for cell survival in *Drosophila* wing pouch cells

**DOI:** 10.1038/s41598-017-10550-4

**Published:** 2017-09-12

**Authors:** Juan Lu, Dan Wang, Jie Shen

**Affiliations:** Department of Entomology, MOA Key Laboratory for monitoring and green management of crop pests, China Agricultural University, 100193 Beijing, China

## Abstract

An appropriate balance between cell survival and cell death is essential for correct pattern formation in the animal tissues and organs. Previous studies have shown that the short-range signalling molecule Hedgehog (Hh) is required for cell proliferation and pattern formation in the *Drosophila* central wing discs. Signal transduction by one of the Hh targets, the morphogen Decapentaplegic (Dpp), is required for not only cell proliferation, but also cell survival in the pouch cells. However, Hh function in cell survival and cell death has not been revealed. Here, we found that loss of Hh signal activity induces considerable Caspase-dependent cell death in the wing pouch cells, and this process was independent of both Dpp signalling and Jun-N-terminal kinase (JNK) signalling. Loss of Hh induced activation of the pro-apoptotic gene *hid* and inhibition of *diap1*. Therefore, we identified an important role of Hh signalling in cell survival during *Drosophila* wing development.

## Introduction

The balance between cell death and cell survival is essential for the development of animal tissues and organs. The disturbance of this balance by massive cell death can result in a great deal of cell loss and can cause developmental defects and diseases^1^. The lack of survival factors results in ectopic apoptosis and further induces tissue abnormalities.

The cell death pathway is highly conserved across animal species^2, 3^. Apoptosis, also known as Programmed Cell Death (PCD), is conducted through a strictly regulated progress^4^. Various types of stimulation, such as X-ray irradiation, mechanical stress and genetic variations, can induce cell death by inducing the expression of pro-apoptotic genes, *reaper* ^5^, *hid* ^6^, and *grim* ^7^ (RHG proteins), and finally by activating Caspases which degrade cellular substrates. There are 7 caspase genes in Drosophila^8^, divided into two classes: the initiator caspases and effector caspases. The effector caspases Drice^9^ and Dcp1^10^ are activated by the initiator caspase Dronc^11, 12^. Caspases are repressed by Inhibitor of Apoptosis Proteins (Diap1) in the absence of cell death stimulation^13–15^. In the presence of a cell death stimulus, Diap1 is inhibited by RHG proteins. The pan-caspase inhibitor P35 can specifically block the function of the effector caspases Drice and Dcp-1 without affecting the activity of the initiator caspase Dronc^16^.

The morphogen Decapentaplegic (Dpp) is required for the cell survival to ensure normal tissue morphology by extruding or degrading the damaged cells^17, 18^. Dpp is expressed in a stripe abutting the A/P compartment boundary and forming a precise concentration gradient along the A/P axis^19–22^. Dpp binds and activates the receptor complex Thickvein (Tkv)/Punt (Put), which phosphorylates Mad to PMad^23^. PMad, together wigh Medea (Med), enters the nucleus and regulate the target genes expression, including *sal* ^24^ and *omb* ^25–27^. One target of PMad, Daughter against dpp (Dad), can regulate Dpp signalling activity via negative feedback^28–30^. The continuous gradient of Dpp signalling activity is required for the cell survival. Sharp discontinuity of either Dpp signalling or Dpp targets can induce JNK-dependent apoptosis which results in aberrant morphogenesis^17, 18, 26^. JNK, encoded by *basket (bsk)* ^31, 32^ and activated by the MAP kinase kinase Hemipterous (Hep)^33^, is involved in apoptotic signalling in various tissues.

Dpp is one of the targets of Hedgehog (Hh) which has been considered as a short-range signal^34–37^. Hh plays a crucial role in proliferation and pattern formation in the central *Drosophila* wing disc^38–41^. The components of Hh were initially identified in *Drosophila* and are conserved in mammals^42^. In *Drosophila* wing disc, Hh is expressed in the posterior compartment and secreted into anterior compartment^43^. The transportation of Hh from posterior to anterior compartment requires Tout-velu (Ttv)^44, 45^. In anterior compartment, Hh binds to receptor Patched (Ptc) to derepress the activity of a transmembrane protein Smoothened (Smo)^44, 46, 47^. The activated Smo maintains Cubitus interruptus (Ci) in an active form^48^. The Ci[act] enters the nucleus and induces target genes expression, including *engrailed* (*en*), *ptc*, *Collier* (*col*), and *dpp*. These target genes are activated in a Hh-concentration dependent manner: The cells close to the AP compartment boundary receive the highest level of Hh and induce the *ptc* and *en*; the cells away from the AP compartment boundary will receive the lowest level of Hh and induce the expression of *col*; the cells between these two type of cells receive the moderate level of Hh and induce the expression of *dpp* ^49–52^. *ptc* acting as the target gene of Hh signaling also inhibits Smo expression in the absence of Hh^46^.

Previous studies have demonstrated that Hh plays an important role in the proliferation^38–40^ and patterning^41, 53–55^. Hh also controls cell survival in germ cells^56, 57^, neural crest cells^58, 59^ as well as tumor cells^60, 61^ in vertebrate. A recent study has shown that in *Drosophila* eye disc, deregulated Hh signalling promotes cell survival in a non-autonomous manner^62^. However, it is not clear whether Hh signalling is also involved in the control of cell survival in wing disc. Here, we found that Hh signaling plays an important role in the cell survival in the *Drosophila* wing pouch. Lacking Hh signaling induced cell death is independent of Dpp and JNK signaling pathways.

## Results and Discussion

### Down-regulation of Hh signalling results in apoptosis in *Drosophila* wing disc

**Figure 1:**
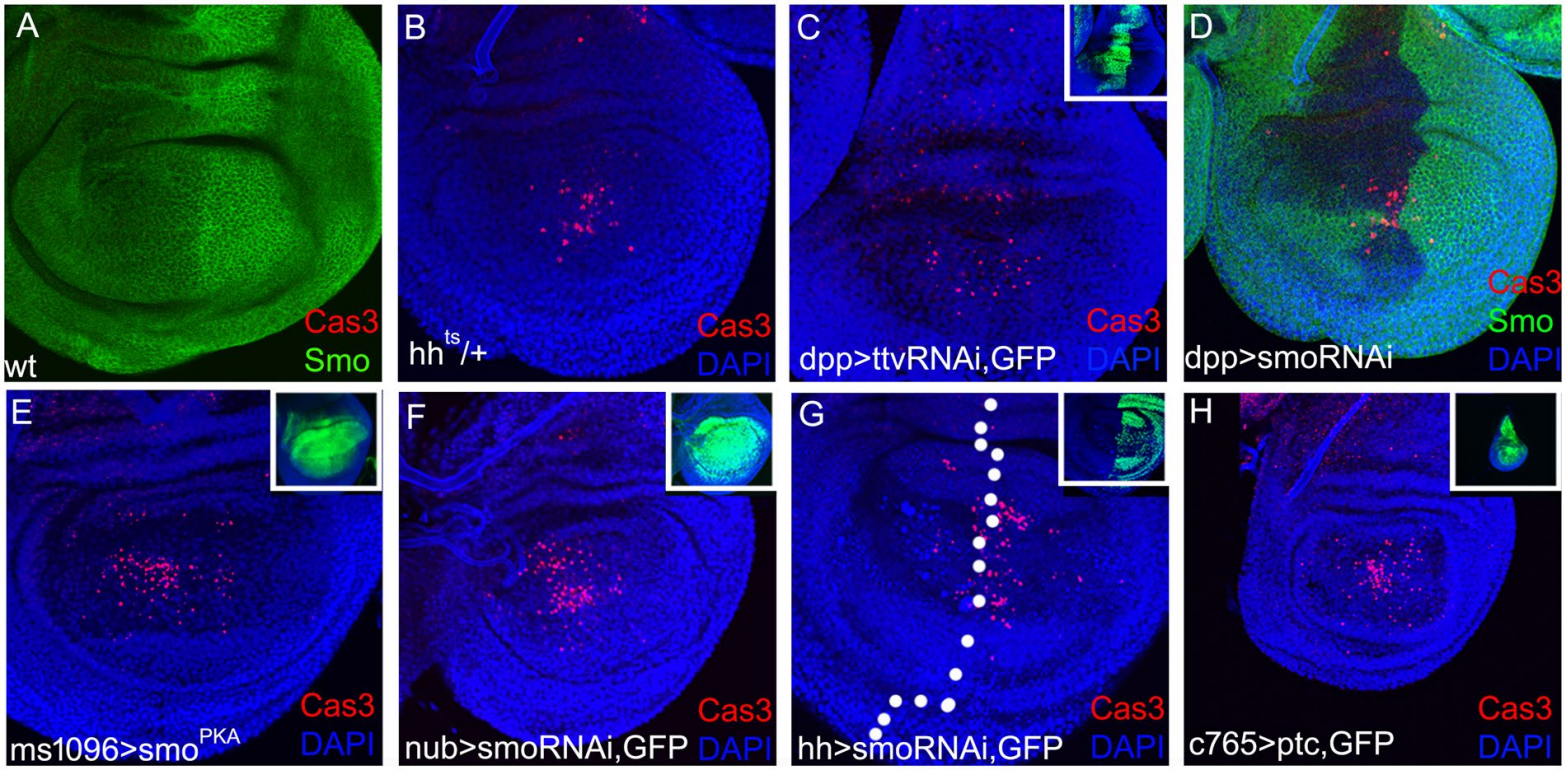
Hh signalling activity is required for cell survival in *Drosophila* wing disc. In this and subsequent figures, wing discs are oriented with dorsal up and anterior left. (**A**) In the wild-type wing disc, there is no obvious apoptosis indicated by anti-Caspase-3 staining (red). The *smo* expression pattern is revealed by anti-Smo staining (green). (**B**) Heterozygote of a hh temperature-sensitive mutant allele showing the induction of apparent cell death (red). (**C**) Suppression of Hh transportation from posterior to anterior by expressing *ttv-RNAi* in the *dpp-Gal4* domain (inset panel, green) results in apparent cell death (red). (**D**) Smo (green) is suppressed by expressing *smo-RNAi* in the *dpp-Gal4* domain, and that induces massive cell death (red). (**E**) Suppressing *smo* by expressing a mutant *smo* ^*PKA*^ in all the wing pouch cells induces massive cell death (red). *ms1096-Gal4* is expressed in all the wing pouch cells with a higher activity within the dorsal compartment (See inset panel, green). (**F** and **G**) Suppressing *smo* by expressing *smo-RNAi* in large regions induces massive cell death (red). The *nub-Gal4* domain covers the pouch region (See inset panel in F, green). *hh-Gal4* is expressed only within the posterior compartment (See inset panel in G, green). (**H**) Suppressing Smo activity by expressing the inhibitor gene *ptc* in all the wing cells under the *c765-Gal4* driver results in small wing discs with massive cell death (red). Note that panel H is also from a 3^rd^ instar larvae and is shown at the same magnification with other pannels. When *ptc* is expressed in the whole wing disc, the wing disc size is reduced apparently due to a proliferation defect.

The wild-type wing disc undergoes rapid proliferation with little apoptosis (Fig. 1A). When down-regulating Hh expression using a temperature-sensitive allele, hh^ts^ ^46^, apoptosis, indicated by anti-Caspase-3 staining, occurred in the wing pouch (Fig. 1B). Then, the Hh transportation from the posterior to the anterior was blocked by expressing *ttv-RNAi* in the *dpp-Gal4* domain, obvious apoptosis was consistently observed in the central wing discs (Fig. 1C). Then, we assessed whether Smo mediates the role of Hh in regulating apoptosis. Apparent apoptosis was also induced in the central wing discs when *smo* was inhibited by the expression of *smo-RNAi* in the *dpp-Gal4* domain (Fig. 1D). To further confirm the above results, Hh signalling activity was suppressed by expressing *smo* ^*PKA12*^ (a mutation at the PKA site)^35^, *smo-RNAi*, and *ptc* in all the wing disc cells (driven by *c765-Gal4*), wing pouch cells (driven by *ms1096-Gal4* and *nub-Gal4*), and posterior cells (driven by *hh-Gal4*). All these manipulations caused obvious apoptosis in the medial wing discs (Fig. 1E–H). These data suggests that suppression of Hh singling, at the levels of transcription, transportation, or signal transduction, induces cell death in the medial *Drosophila* wing disc, thereby revealing a new role for Hh signalling in cell survival.

### Apoptosis induced by the lack of Hh signalling is Dpp-independent

**Figure 2:**
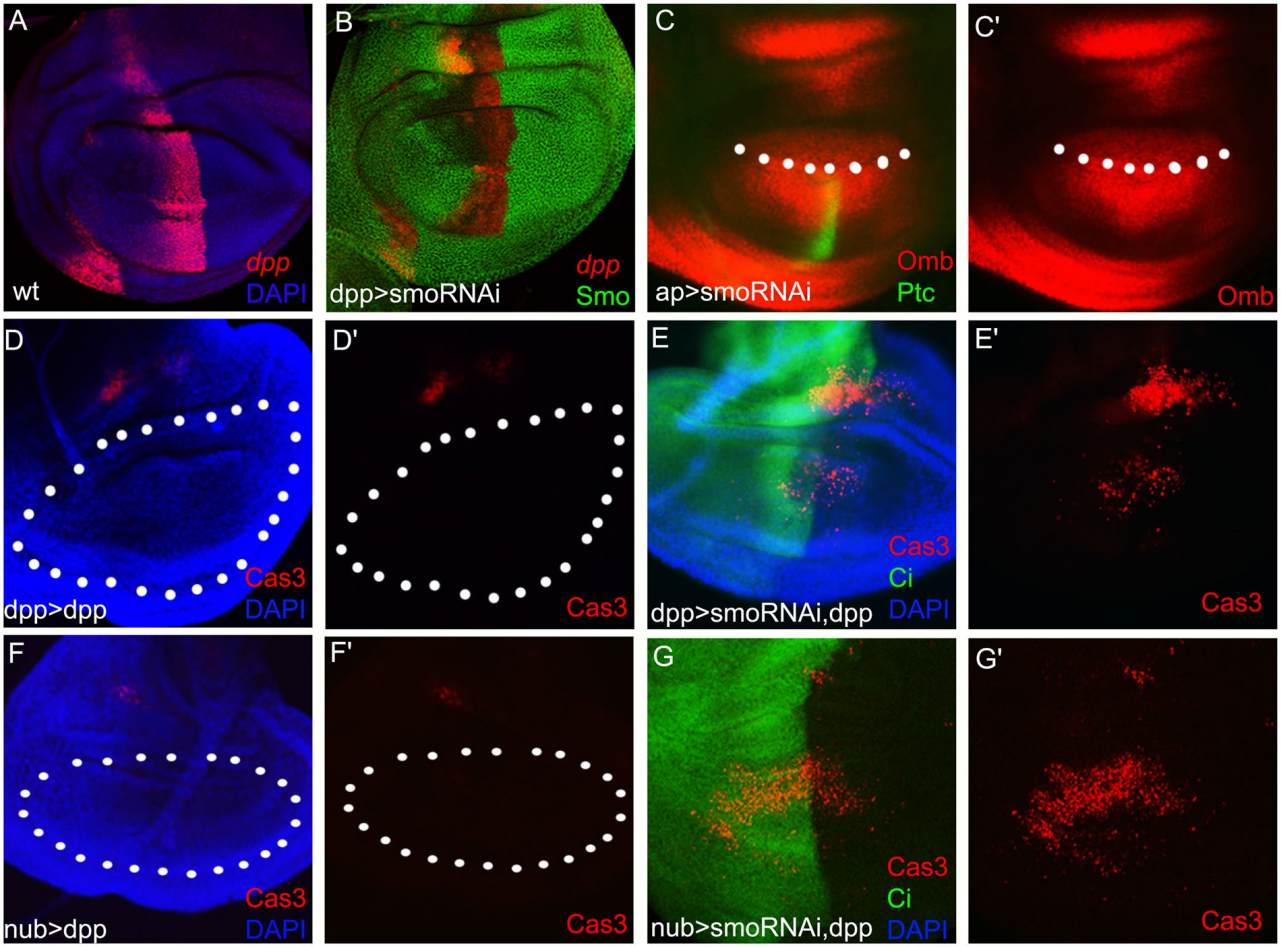
Cell death caused by Hh signalling reduction is dpp-independent. (**A**) The *dpp* expression pattern in wild-type wing discs is revealed by the *dpp-lacZ* enhancer trap line (red). (**B**) *dpp-lacZ* is still present (red) when Smo (green) is suppressed within the *dpp-Gal4* domain. (**C**) Omb (red) is still detectable when *smo* is inhibited in the dorsal compartment by the dorsal-specific driver *ap-Gal4*. The Hh target Ptc is apparently inhibited within the dorsal compartment (green). The dotted line indicates the boundary between the dorsal and ventral compartments. (**D** and **F**) In control experiments, *dpp* is expressed within the *dpp-Gal4* domain (**D**) and the *nub-Gal4* domain (**F**). No cell death occurs within the wing pouch regions (dotted regions) except for a patch of dead cells (red) in the presumptive hinge domains, which might be a side effect of overgrowth induced by excess Dpp. (**E** and **G**) The apoptosis (red) is still induced even when *dpp* is co-expressed with *smo-RNAi* in the *dpp-Gal4* (**E**) and *nub-Gal4* (**G**) domains. A specific marker for the anterior compartment, Ci, is revealed by anti-Ci staining (green), to show the midline of overgrown wing discs.

*dpp*, one target gene of Hh signalling, has been demonstrated to be an important survival factor^24–26^. To test whether the apoptosis caused by suppression of Hh signalling is due to the reduction of Dpp signalling, we examined the *dpp* expression using a *dpp-lacZ* reporter. In the wild-type background, *dpp* is expressed in a stripe of cells along the AP boundary (Fig. 2A). When Hh signalling was suppressed by *smo-RNAi*, the *dpp* transcription level was mildly reduced compared with that in wild type (Fig. 2A and B). Ptc, which is only expressed in a narrow stripe of cells just anterior to the AP compartment boundary by sensing the highest level of Hh, is a direct readout of Hh signalling. To obtain an internal control, we used a dorsal-specific driver, *ap-Gal4*, to express *smo-RNAi* (Fig. 2C). Ptc was abolished completely in the *ap-Gal4* region (Fig. 2C), while Omb, one of the targets of Dpp signalling, was still detectable. The apoptosis was consistently observed in the *ap > smoRNAi* wing disc (Fig. S1). These data implied that the cell death might be a direct consequence of the suppression of Hh signalling and not a side effect of the reduction in Dpp signalling. To test this possibility, we co-expressed *dpp* with *smo-RNAi* to see whether the apoptosis can be rescued. In the control, *dpp* was solely expressed in either the *dpp-Gal4* or the *nub-Gal4* region, and there was no cell death in the pouch region except in the notum region (Fig. 2D and F). When *dpp* was co-expressed with *smo-RNAi* in the *dpp-Gal4* domain, the apoptosis was still present in the wing pouch (Fig. 2E). The failure of *dpp* in the rescue experiment was confirmed in the *nub-Gal4* domain (Fig. 2G). Taken together, the cell death caused by the suppression of Hh signalling is a direct consequence of the Hh pathway and not a side effect of disturbance in Dpp signalling.

### Cell death induced by the lack of Hh signalling is JNK independent

**Figure 3:**
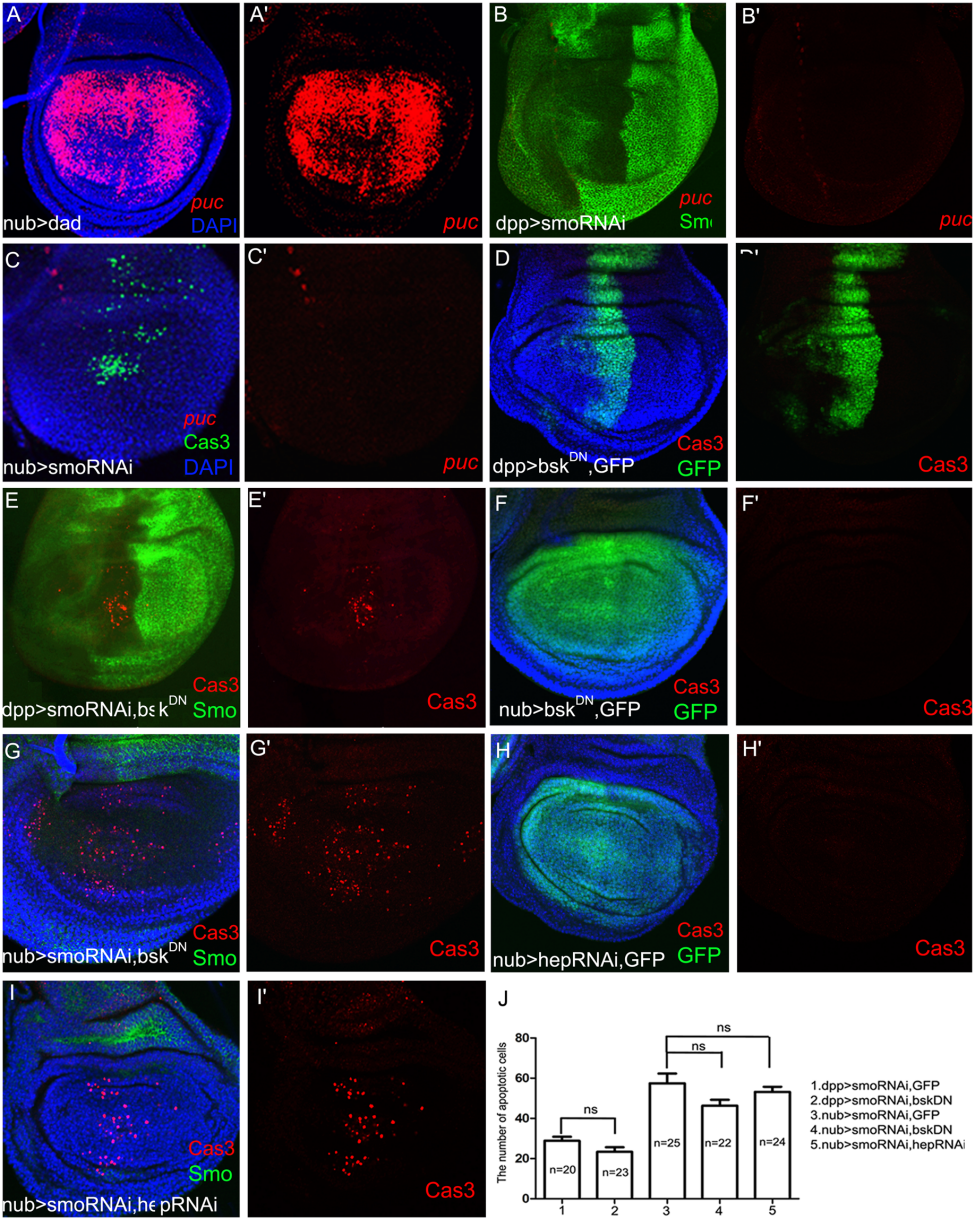
The cell death induced by suppressing Hh signalling is JNK-independent. (**A**) Ectopic JNK signalling activity can be revealed by using a *puc-lacZ*. In a positive control experiment, *puc-lacZ* reporter (red) is activated when Dpp signalling is suppressed by expressing *dad* within the *nub-Gal4* domain. (**B**,**C**) Suppression of Hh signalling by expressing *smo-RNAi* does not induce ectopic *puc-lacZ* (red). (**D**–**I**) Inhibition of JNK signalling by expressing a dominant negative form of *bsk* ^*DN*^ (**E** and **G**) or by suppressing an upstream effector (by expressing *hep-RNAi*) (**I**) can not rescue the apoptosis induced by expressing *smo-RNAi*. (**D**,**F** and **H**) The control experiments show no apoptosis when *bsk* ^*DN*^ or *hep-RNAi* is expressed alone. (**J**) Statistics for the apoptotic cell number per wing disc of each genotype mentioned above. ns stands for no significant difference.

Previous studies have shown that JNK signalling plays a vital role in cell morphology, cell invasion, and apoptosis^26, 31, 63, 64^. JNK, monitored by *puc-lacZ* ^65^, was activated when Dpp signalling was inhibited by expressing its inhibitor *dad* (Fig. 3A). However, there was no ectopic *puc-lacZ* expression when Hh signalling was inhibited (Fig. 3B and C). Furthermore, the apoptosis was not reduced when JNK signalling was inhibited by co-expressing a dominant negative form of *bsk* (*bsk* ^*DN*^) (Fig. 3D,E,F,G and J) or by co-expressing *hep-RNAi* (Fig. 3H,I and J). These data suggested that the apoptosis caused by the suppression of Hh signalling is independent of JNK signalling.

### Cell death induced by the lack of Hh signalling is mediated by *hid* and *diap1*

**Figure 4:**
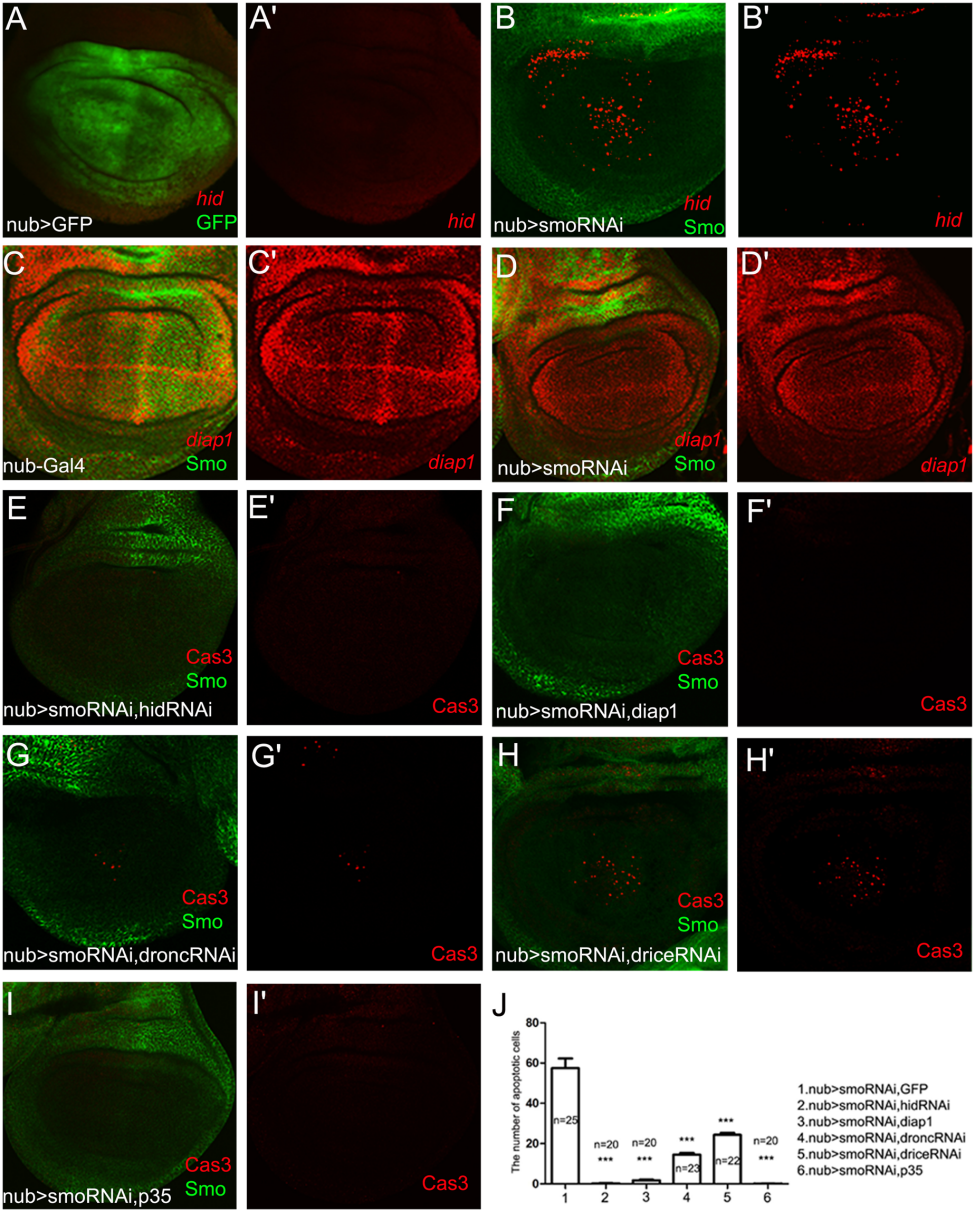
Cell death induced by the lack of Hh signalling activity requires the activation of the pro-apoptotic gene *hid* and the reduction of *diap1*. (**A**) In the control *nub > GFP* wing disc, there is no apparent *hid-lacZ* expression (red). (**B**) The pro-apoptotic gene *hid* (red) is activated in the wing pouch when *smo-RNAi* is expressed within the *nub-Gal4* domain. (**C**,**D**) *diap1-lacZ* (red) is apparently reduced in the medial region of the *nub > smo-RNAi* wing discs (**D**) compared with the control (**C**). (**E**) Suppressing pro-apoptotic gene *hid* by expressing *hid-RNAi* efficiently suppresses the cell death compared with the control (Fig. 1F). (**F**) Co-expression of *diap1* with *smo-RNAi* efficiently suppresses the cell death. (**G**) Suppressing initiator caspase activity by expressing *dronc-RNAi* largely suppresses the cell death induced by *smo-RNAi* expression. (**H**) Suppressing effector caspase activity by expressing *drice-RNAi* reduces the cell death induced by *smo-RNAi* expression. (I) Co-expression of *P35* with *smo-RNAi* suppresses the cell death completely. (**J**) Statistical analysis for the apoptotic cell number per wing disc of each genotype mentioned above. Means ± SEM indicated *** are significantly different (pairwise comparisons performed using t-tests, p < 0.0001).

Apoptosis is a highly conserved pathway in both invertebrate and vertebrate systems. The key mediators, including Hid, Drice, Dronc, and Diap1, were mentioned in the introduction section. Subsequently, we tested whether there was a link between the cell death pathway and the Hh pathway. When *smo* was suppressed in the wing pouch, the transcription of *hid-lacZ* was markedly increased (Fig. 4A and B), while the transcription of *diap1-lacZ* was reduced (Fig. 4C and D). Co-express *hid-RNAi* and *smo-RNAi* in the nub-Gal4 domain supppressed the cell death (Fig. 4E and J). The cell death was also suppressed completely when *diap1* was co-expressed with *smo-RNAi* in the *nub-Gal4* domain (Fig. 4F and J). Next, we examined the roles of an initiator caspase (Dronc) and an effector caspase (Drice). Use of either *dronc-RNAi* or *drice-RNAi* partially rescued the apoptosis (Fig. 4G,H and J) compared with the control (Fig. 1F). This cell death was suppressed completely when P35 was co-expressed with *smo-RNAi* in the *nub-Gal4* domain (Fig. 4I and J). Taken together, we demonstrated that the cell death caused by the suppression of Hh signalling is at least partially mediated by the activation of the proapoptotic gene *hid* and by inhibition of *diap1*.

### Cell death induced by the lack of Hh signalling led to small adult wings

**Figure 5:**
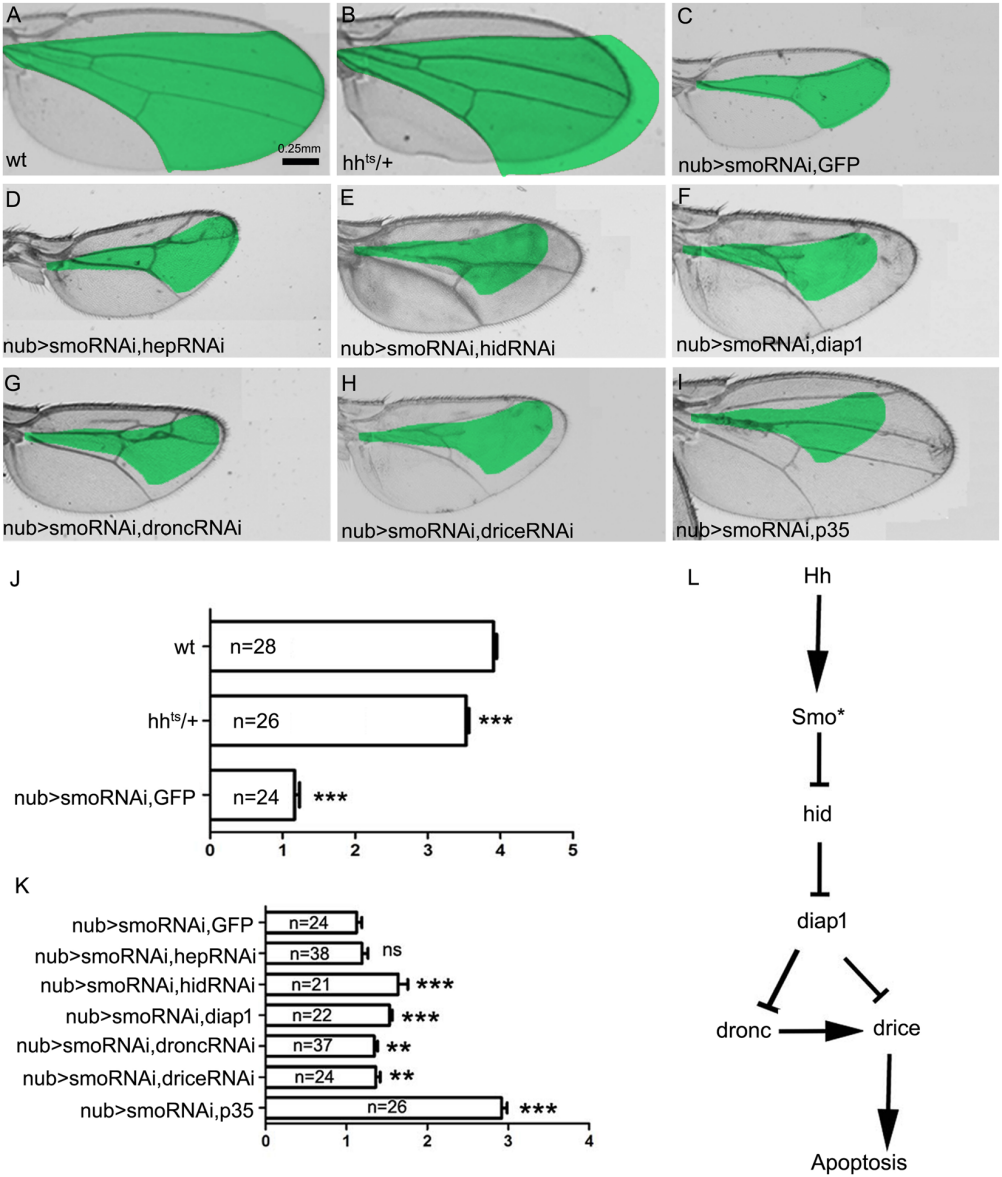
The phenotypes of adult wings. (**A**) Wild-type adult wing. The area between L2 and L5 veins is measured. The region between L2 and L5 veins of each control wing (**A** and **C**) is marked in green and compared in each manipulation (**B**,**D**–**H**) because most of the cell death induced by the suppression of Hh signalling occurs in the presumptive region between the L2 and L5 veins. (**B**) The *hh* ^*ts*^ adult wing is smaller than the wild-type wing (**A**). (**D**) Co-expressing *hep-RNAi* does not rescue the small size between L2 and L5 compared with the expression of *smo-RNAi* (**C**). (E) Co-expressing *hid-RNAi* increases the indicated area compared with the control wing in C. (**F**) Co-expressing *diap1* increases the indicated area compared with the control wing in C. (**G**) Co-expressing dronc-RNAi increases the indicated area compared with the control wing in C. (**H**) Co-expressing *drice-RNAi* increases the indicated area compared with the control wing in C. (**I**) The wing area is rescued by co-overexpressing *P35*. (**J** and **K**) Statistics for the green-marked wing regions in each genotype mentioned above. Means ± SEM indicated by ** or *** are significantly different (pairwise comparisons performed using t-tests, p < 0.001 or p < 0.0001). (**L**) A model of the genetic pathway regulating cell survival by Hh signalling.

To assay the apoptosis effect on adult wing, we measured the size of the medial wing where apoptosis always occur in the manipulations of Hh signalling. Compared with the wild-type adult wing (Fig. 5A), reduction of Hh signalling by hh^ts^ (Fig. 5B and J) and smo-RNAi (Fig. 5C and J) in the whole wing blade resulted in an obvious reduction in wing size. There was no significant difference in adult wing size between *nub > smo-RNAi*, GFP and *nub > smo-RNAi, hep-RNAi* (Fig. 5C, D and K). Suppression of the pro-apoptotic genes *hid* by *hid-RNAi* showed rescue effect in adult wing size (Fig. 5C,E and K). Co-expressing *diap1* with *smo-RNAi* in the *nub-Gal4* domain had an obvious rescue effect of adult wing size compared with *smo-RNAi* alone (Fig. 5C,F and K). However, the adult wing size of *nub > smo-RNAi, hid-RNAi* and *nub > smo-RNAi, diap1* did not restore to the wild type size, which may be due to the proliferation effect of Hh signalling. Suppression of the initiator caspase and the effector caspase showed a slight rescue effect in adult wing size (Fig. 5C,G,H and K). Co-expressing the pan-caspase inhibitor P35 could largely rescue the adult wing size (Fig. 5I and K). These data suggest that the wing size is regulated not only by proliferation control, but also by cell survival control of Hh signalling.

The wing veins’ pattern was also altered. Consistent with previous reports, lacking Hh signalling lead to loss of L3 and L4 veins (Fig. 5C)^41, 55, 66^. When the cell death was suppressed by *hid-RNAi* or* d* *ronc-RNAi*, the L4 vein was rescued (Fig. 5E and G). When the cell death was suppressed by *diap1* or *drice-RNAi*, the L3 and L4 veins were only partially rescued up to the proximal region with fusion effect (Fig. 5F and H). The L3 and L4 veins could be completely rescued only when the cell death is suppressed by P35 (Fig. 5I). Therefore, Hh signalling regulates the medial wing pattern formation, at least in part, by control of cell survival.

Various signalling pathways are involved in cell survival. The Hippo/Warts/Yorkie (Hpo/Wts/Yki) pathway is known to control apoptosis. Hpo negatively regulates the transcription factor Yki by phosphorylating it. The dephosphorylation of Yki activates the target gene *diap1* to inhibit apoptosis^67–69^. Notch and Wingless (Wg) promote cell survival by inhibiting Caspase^70–72^. Epidermal Growth Factor Receptor (EGFR) is required for cell survival in the *Drosophila* eye disc, where it inhibits the pro-apoptotic gene *hid* ^73–75^. Dpp is involved in cell survival by activating the downstream target genes *omb* ^24^ and *sal* ^25–27^. Here, we found that Hh is also involved in cell survival in the *Drosophila* wing disc through *hid* and *diap1*, and we present a model to explain the possible genetic regulation (Fig. 5L). Although *hid-RNAi* and *diap1* can efficiently suppress the cell death induced by *smo-RNAi* expression (Fig. 4E,F and J), the adult wings are not restored to wild type size (Fig. 5E,F and K). We can not rule out a possibility of compensational mechanism between the Hh-regulated cell survival and proliferation. However, the disruption of any of the above signalling pathways can induce apoptosis. There must be a mechanism by which a cell integrates all of these signals to determine its survival status. Our results suggest that Smo is the most downstream component of Hh signalling that is related to cell survival. To better understand how Hh-Smo signalling promotes cell survival, the potential mediators between Smo and apoptosis pathway need to be identified. In the *Drosophila* eye, *diap1* is up-regulated by deregulated Hh signalling^62^. In the *Drosophila* wing, *diap1* is apparently suppressed under the condition of Hh loss-of-function (Fig. 4D and D’). Hh has been reported essential for the cell survival in vertebrate^56–61^. A recent study has shown that the requirement of Hh in cell survival in pancreatic cancer cells is dependent on the up-regulation of baculoviral IAP repeat-containing 3 (BIRC3) gene which belongs to IAP family^76^. Therefore, Hh is functionally conserved in cell survival control in both vertebrate and invertebrate.

## Materials and methods

### Drosophila stocks

The following transgenes were used: *dpp-Gal4* ^77^, *ms1096-Gal4* ^78^, *nub-Gal4* ^79^, *hh-Gal4* (BL#45169), *c765-Gal4* ^80^, *UAS-ttvRNAi* (VDRC#4871), *UAS-smoRNAi* (VDRC#9542), *UAS-smo* ^*PKA*35^, *UAS-ptc* (BL#5817), *UAS-dpp* (BL#1486), *UAS-diap1* ^81^, *UAS-droncRNAi* ^82^, *UAS-driceRNAi* (VDRC#28065), *UAS-P35* (BL#5073), *UAS-dad* ^28^, *UAS-bsk* ^*DN*^ (BL#6409), *UAS-hepRNAi* (VDRC#47507), *UAS-hidRNAi* (a gift from Lei Xue). Mutant alleles used were: *hh* ^*ts*^ (BL#1684). Enhancer trap lines used were: *dpp-lacZ* ^83^, *puc-lacZ* (BL#11173), *hid-lacZ* ^84^, *diap1-lacZ* ^85^. Larvae were raised at 25 °C unless stated otherwise. For efficient expression of RNAi transgenes, larvae were raised at 29 °C.

### Dissection of larvae

Wing imaginal discs were dissected from 3^rd^ instar *Drosophila* larvae according to a standard protocol and were fixed for 30 min in 4% paraformaldehyde in PBT (PBS with 0.3% Triton X-100).

### Immunohistochemistry

Fixed wing imaginal discs were stained with antibodies according to standard procedures. The primary antibodies used were: rabbit anti-Caspase3, 1:200 (Cell Signaling Technology); mouse anti-β-galactosidase, 1:2000 (Promega); rabbit anti-β-galactosidase 1:2000 (Promega); rat anti-Ci, 1:200 (DSHB); mouse anti-En, 1:200 (DSHB); mouse anti-Smo, 1:200 (DSHB); mouse anti-Ptc, 1:200 (DSHB). Secondary antibodies used were goat anti-mouse DyLight 549, goat anti-rat DyLight 549, and goat anti-rabbit DyLight 488, 1:200 (Agrisera). Images were collected using a Leica TCS-SP2-AOBS confocal microscope.

### Adult wing imaging

Adult wing images were collected using an inverted microscope (AMG EVOS, America).

### Adult wing measurement

The area of the adult wing was measured using Image-J software, and the calculation and measurement were carried out using GraphPad Prism 5 Project.

## Electronic supplementary material

Supplementary Information
