# Supplementary material for: Hedgehog signalling is required for cell survival in Drosophila wing pouch cells
Source: Sci Rep. 2017 Sep 12;7:11317. doi: 10.1038/s41598-017-10550-4 (PMC5595820; doi:10.1038/s41598-017-10550-4)
Supplement: Supplementary file 1 — Supplementary Information [file 41598_2017_10550_MOESM1_ESM.pdf]

Hedgehog signalling is required for cell survival in *Drosophila* wing pouch cells

Juan Lu, Dan Wang and Jie Shen*
Department of Entomology, MOA Key Laboratory for monitoring
and green management of crop pests, China Agricultural University, 100193 Beijing, China
*Corresponding author. E-mail: shenjie@cau.edu.cn, Tel (0086) 10 627 32384

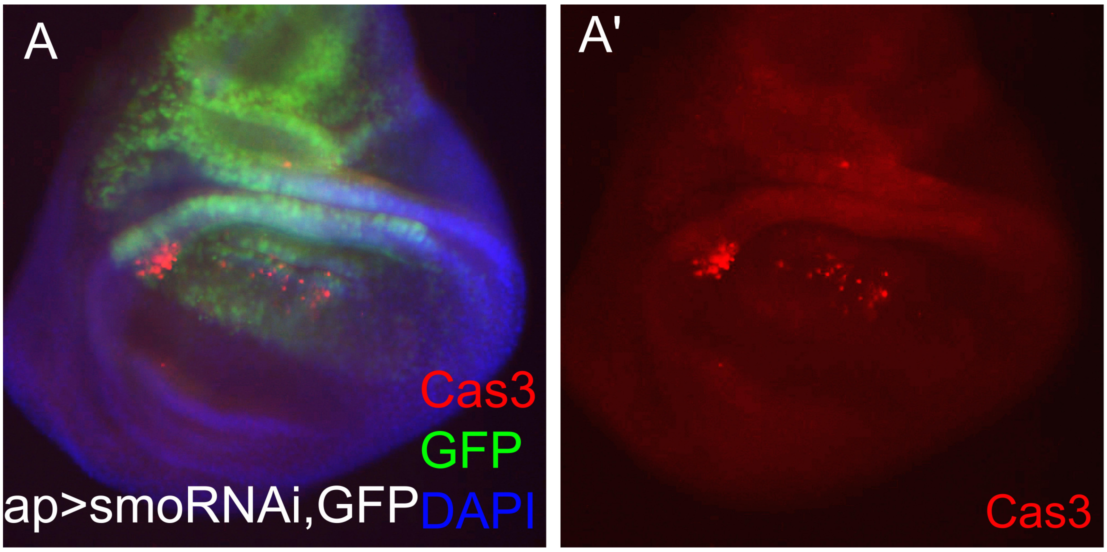

Suppression of *smo* by expressing *smo-RNAi* induced apoptosis in the ap-Gal4 domain.
